# Genome-wide analysis of DNA methylation in bronchial washings

**DOI:** 10.1186/s13148-018-0498-8

**Published:** 2018-05-18

**Authors:** Sang-Won Um, Yujin Kim, Bo Bin Lee, Dongho Kim, Kyung-Jong Lee, Hong Kwan Kim, Joungho Han, Hojoong Kim, Young Mog Shim, Duk-Hwan Kim

**Affiliations:** 1 Department of Internal Medicine, Samsung Medical Center, Sungkyunkwan University School of Medicine, Seoul, 135-710 South Korea; 2 Department of Molecular Cell Biology, Samsung Biomedical Research Institute, Sungkyunkwan University School of Medicine, Suwon, 440-746 South Korea; 3 Department of Thoracic and Cardiovascular Surgery, Samsung Medical Center, Sungkyunkwan University School of Medicine, Seoul, 135-710 South Korea; 4 Department of Pathology, Samsung Medical Center, Sungkyunkwan University School of Medicine, Seoul, 135-710 South Korea; 5 Samsung Medical Center, Research Institute for Future Medicine, #50 Ilwon-dong, Kangnam-gu, Professor Rm #5, Seoul, 135-710 South Korea

**Keywords:** Hypermethylation, Lung cancer, Smoking, Bronchial washing, Epigenome

## Abstract

**Background:**

The objective of this study was to discover DNA methylation biomarkers for detecting non-small lung cancer (NSCLC) in bronchial washings and understanding the association between DNA methylation and smoking cessation.

**Methods:**

DNA methylation was analyzed in bronchial washing samples from 70 NSCLCs and 53 hospital-based controls using Illumina HumanMethylation450K BeadChip. Methylation levels in these bronchial washings were compared to those in 897 primary lung tissues of The Cancer Genome Atlas (TCGA) data.

**Results:**

Twenty-four CpGs (*p* < 1.03E−07) were significantly methylated in bronchial washings from 70 NSCLC patients compared to those from 53 controls. The CpGs also had significant methylation in the TCGA cohort. The 123 participants were divided into a training set (*N* = 82) and a test set (*N* = 41) to build a classification model. Logistic regression model showed the best performance for classification of lung cancer in bronchial washing samples: the sensitivity and specificity of a marker panel consisting of seven CpGs in *TFAP2A*, *TBX15*, *PHF11*, *TOX2*, *PRR15*, *PDGFRA*, and *HOXA11* genes were 87.0 and 83.3% in the test set, respectively. The area under the curve (AUC) was equal to 0.87 (95% confidence interval = 0.73–0.96, *p* < 0.001). Methylation levels of two CpGs in *RUNX3* and *MIR196A1* genes were inversely associated with duration of smoking cessation in the controls, but not in NSCLCs, after adjusting for pack-years of smoking.

**Conclusions:**

The present study suggests that NSCLC may be detected by analyzing methylation changes of seven CpGs in bronchial washings. Furthermore, smoking cessation may lead to decreased DNA methylation in nonmalignant bronchial epithelial cells in a gene-specific manner.

**Electronic supplementary material:**

The online version of this article (10.1186/s13148-018-0498-8) contains supplementary material, which is available to authorized users.

## Background

Lung cancer is the most common cause of cancer deaths worldwide. Despite recent advances in the diagnosis and treatment for lung cancer, prognosis of patients remains very poor. The overall 5-year survival rate of lung cancer has improved slightly from 12 to 16% over the past 30 years [1]. Such poor prognosis is largely due to occult metastatic dissemination of tumor cells, which occurs in more than half of all patients at the time of diagnosis. The majority of patients undergoing curative surgical resection at an early stage have achieved long-term survival. Overall 5-year survival rate for patients with surgically resected stage IA, stage IB, and stage II non-small cell lung cancers (NSCLCs) has been reported to be 83, 69, and 48%, respectively [2]. Accordingly, it is imperative to develop efficient diagnostic tools that can identify lung cancer at an early stage so that curative treatment is feasible.

De novo methylation of CpG islands at the promoter region of tumor suppressor genes is usually associated with transcriptional silencing of a gene. It is one of the most common epigenetic modifications in lung cancer. Aberrant methylation of CpG loci in bronchial washings could become a powerful tool for early diagnosis of lung cancer. To discover aberrant methylation that occur at an early stage of lung cancer, several groups have analyzed methylation statuses of multiple CpG loci in bronchial aspirate or sputum from both lung cancer patients and healthy individuals [3–9]. Epigenetic studies of human cancer have recently shifted from candidate gene analyses toward epigenome-wide analysis with rapid technological advances. However, most studies on bronchial aspirate were restricted to a few candidate CpG loci with inadequate genome coverage.

Cigarette smoke is a well-known environmental modifier of DNA methylation [10]. It modulates DNA methylation by the following these mechanisms: (i) recruiting DNA methyltransferase 1 (DNMT1) to damage sites during DNA repair; (ii) altering nuclear protein levels and activity of DNA-binding factors such as SP1; or (iii) inhibiting GSK3β function and attenuating DNMT1 degradation [11–13]. Recently, several groups have reported a reversibility of methylation change after smoking cessation in peripheral blood samples. Ambatipudi et al. [14] have reported that methylation levels at smoking-related CpG sites are reversible after smoking cessation, although changes in DNA methylation of specific genes can remain for up to 22 years after quitting smoking. Guida et al. [15] have also reported that some methylated CpGs can revert back to levels present in never-smokers after a certain time since smoking cessation.

In this study, we analyzed DNA methylation at the genome level to discover a panel of CpGs for the detection of NSCLC in bronchial washings and investigated the effect of smoking cessation on reversion of methylated DNA to normal levels.

## Methods

### Study population

A total of 123 patients (70 non-small cell lung cancers [NSCLCs] and 53 hospital-based controls with benign lung disease) who were admitted for fiberoptic bronchoscopy or for curative surgical resection at Samsung Medical Center in Seoul, Korea, between March 2010 and November 2015 participated in this study. NSCLC patients underwent surgery as well as bronchoscopy. Controls were recruited from patients with benign lung diseases such as actinomycosis, anthracofibrosis, bronchiolitis, pneumonia, or tuberculosis. Patients with benign lung tumors such as localized organizing pneumonia or hamartoma were excluded from this study. This is because methylation profiling of these diseases is not well known yet which can lead to misclassification. Small-cell lung cancer was also excluded from this study for comparison with The Cancer Genome Atlas (TCGA) methylation profiling. Stage IIIB and IV NSCLCs were excluded because this study was aimed to identify biomarkers for detecting NSCLC at early stages.

Bronchial washings were obtained through bronchoscopy with written informed consent from all participants. The control group received bronchoscopy to confirm the diagnosis. Bronchial washing samples were analyzed cytologically for the presence of malignant cells. All lung cancer patients were diagnosed with early-stage lung cancer which was pathologically proven. They underwent curative resection. Information on sociodemographic characteristics was obtained through an interviewer-administered questionnaire. Individuals who were clinically negative for cancer at the time of bronchoscopy without having abnormal findings on their chest radiograph or chest computerized tomography (CT) were included in the hospital-based control group. This study was approved by the Institutional Review Board (IRB #: 2010-07-204) of Samsung Medical Center. All cases of NSCLC were classified based on the guideline of tumor-node-metastasis (TNM) staging system introduced by the American Joint Committee on Cancer [16].

### Bronchoscopy

Flexible fiberoptic bronchoscopy (Olympus, Tokyo, Japan) and sample preparation were performed as described previously [4]. Bronchial washing was performed by instilling 10 mL of sterile normal saline when the bronchoscope (Olympus, Tokyo, Japan) was located at the segmental bronchi of the pulmonary lobe. Bronchial washing samples were mixed with 100 mg of N-acetylcysteine for 10 min to disrupt disulfide bond in mucoproteins and stored at − 20 °C until use.

### Genome-wide methylation analysis

Genomic DNA was extracted from bronchial washing specimens using a QIAamp DNA Blood Mini Kit (Qiagen, Valencia, CA, USA) according to the manufacturer’s instructions, followed by quality control using a UV spectrophotometer (Pharmacia Biotech, Cambridge, England). Double-stranded DNA in solution was quantitated using PicoGreen™ double-stranded DNA quantitation kit (Molecular Probes, Eugene, OR, USA) on a SpectraMax Gemini UV spectrometer (Molecular Devices, Sunnyvale, CA, USA). Bisulfite treatment of genomic DNA was performed using Zymo EZ DNA Methylation Kit (Zymo Research, Orange, CA, USA). Genome-wide DNA methylation levels were measured using Infinium HumanMethylation450 BeadChip (Illumina, Inc) according to the manufacturer’s instructions. *β* value ranging from 0 (no methylation) to 1 (100% methylation) was measured as the ratio of signal intensity of methylated alleles to the sum of methylated and unmethylated signal intensity of alleles at each CpG site.

### Pyrosequencing

Methylation levels obtained by 450K array were validated by pyrosequencing a cg27364741 locus at the promoter region of *OTX1* gene using QIAGEN’s PyroMark Q24 systems. Biotinylated PCR primer sets for amplification of the locus were purchased from Qiagen (Cat no. PM00616336).

### Feature selection for predicting lung cancer

Preprocessing of 450K array data was conducted using wateRmelon [17] and ran on R programming language. After preprocessing, candidate CpGs for NSCLC prediction were selected in the following order: (i) identifying differentially methylated CpGs; (ii) removing age-related methylation; (iii) performing gene set enrichment analysis; (iv) selecting features; and (v) testing model performance. Gene set enrichment analysis was performed using DAVID (http://david.abcc.ncifcrf.gov/). Annotation clusters with EASE score (a modified Fisher’s exact *p* value) below 1.0E−5 were selected as candidate clusters for model building. Any candidate CpG that was significantly correlated in the same cluster was removed from model building.

### Statistical analysis

*T* test (or Wilcoxon rank-sum test) and chi-square test (or Fisher’s exact test) were used to analyze continuous and categorical variables, respectively. Pearson’s (or Spearman’s) rank correlation coefficient was used to analyze correlations between two continuous variables. Linear regression analysis was performed to analyze the effect of smoking cessation on DNA methylation after adjusting for potential confounding factors such as pack-years of smoking. Multiple logistic regression analysis was performed to discover methylated CpGs associated with the development of NSCLC after controlling for age, sex, and smoking status. Statistical analysis was conducted using R software (version 3.1.1). Diagnostic performance of the model was measured using a receiver operating characteristic (ROC) curve, which was created with MedCalc statistical software (version 16.8).

## Results

### Methylation levels of 450K array in bronchial washings were slightly inflated

Assay quality of 450K array was tested by comparing measured DNA methylation levels with levels of predefined subsets (0, 33, 66, and 100%) that were prepared by mixing fully methylated and unmethylated human control DNA (Qiagen, Hilden, Germany). Methylation levels of predefined subsets were similarly reproduced by 450K array (Additional file 1: Figure S1A). *β* values from 450K array were further confirmed using pyrosequencing (Additional file 1: Figure S1B). A cg27364741 locus at *OTX1* gene that was significantly methylated in bronchial washing showed higher methylation in the 450K array than that in pyrosequencing, suggesting background signal of the 450K array (Additional file 1: Figure S1C). Data from the 450K array were quantile normalized using the wateRmelon R package. Samples were first filtered using the “pfilter” function from the wateRmelon package. Samples having 1% of CpG sites with a detection *p* value > 0.05, CpG sites containing a beadcount < 3 in 5% of samples, and CpG sites with a detection *p* value > 0.05 in 1% of samples were removed. Data preprocessing including background noise removal and type I/type II probes bias correction was conducted using the “dasen” function from the package. A total of 2046 (0.42%) of 485,577 CpGs were filtered out.

### Identification of differentially methylated CpGs in bronchial washing

Clinicopathological characteristics of 53 hospital-based controls and 70 NSCLC patients are described in Additional file 2: Table S1. Average ages of controls and cases were 55 and 64 years, respectively. This difference in age was statistically significant (*p* < 0.0001). The proportion of women was significantly higher in controls than that in cases (45 vs. 26%, *p* = 0.02). The control group had more never-smokers than the case group (55 vs. 29%, *p* = 0.007). This required age- and sex-matched controls with absence of significant differences in smoking. However, we could not match controls by age and sex due to low statistical power. Instead, we stratified these data into cases and controls and analyzed age- and smoking-related methylation separately for cases and controls as different cohorts. To identify differentially methylated CpGs in bronchial washings from 70 cases and 53 controls, we transformed the *β* values of methylation level into log scale (log2[*β*/(1 − *β*)]) because the distribution of *β* values did not follow a normal distribution (Shapiro-Wilk test, *p* < 0.05). It was negatively skewed in 450K data from bronchial washing samples. Fifty-eight CpGs with *p* value less than or equal to 1.03E−07 (Bonferroni significance threshold) in two-sided Student’s *t* test were identified from 483,531 CpGs. We selected 31 (Additional file 3: Table S2) out of these 58 CpGs after removing 27 CpGs with a maximum *β* value greater than 0.3 in 53 control samples because background signal in the control DNA of 0% methylation was mostly below *β* = 0.3. Finally, we applied multiple logistic regression analysis to find CpGs related to lung cancer after adjusting confounding factors such as age, sex, and the pack-years of smoking. Twenty-four CpGs were found to be significantly methylated in bronchial washing from lung cancer patients than those from healthy individuals. Of the 31 CpGs, the 7 CpGs that were not statistically significant in multiple logistic regression were as follows: SLC15A3 and 6 CpGs (*HOXA9*, *EVX1*, *HIST1H2BK*, *EMX1*, *ITPK*, and *PRDM14*) showing age-related methylation (Additional file 4: Figure S2) in 70 NSCLCs and 53 controls.

### Feature selection for lung cancer classification and the performance evaluation of proposed models

**Fig. 1:**
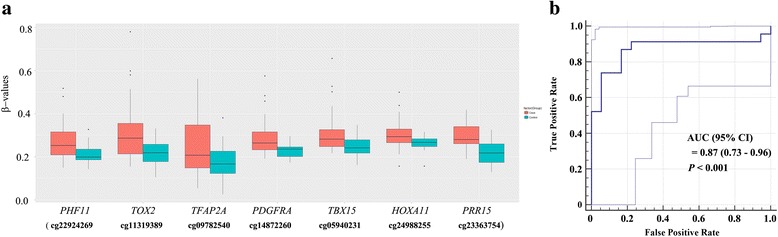
Prediction performance of a panel of seven CpGs. **a** Methylation levels of CpGs on seven genes were compared between 45 NSCLCs and 37 controls in a training set. All CpGs showed significant difference (*p* < 0.05) in *β* values between the two groups. **b** The prediction performance of the seven-CpG panel was evaluated in 41 test samples. The area under curve of the receiver operating characteristic curve in predicting NSCLC using the panel was 0.87 (95% confidence interval: 0.73–0.96; *p* < 0.001). The *X*- and *Y*-axes indicate false positive rate (1 − specificity) and true positive rate (sensitivity), respectively

The 123 samples were divided into a training set (*N* = 82) and a test set (*N* = 41), and the training set was used to build a classification model. Supervised machine learning algorithms, including support vector machine (SVM), random forest, decision tree, artificial neural network (ANN), logistic regression analysis, and K-nearest neighbor, were used to build models. An optimal subset of CpG features for use in building the classification models was selected from the training set with full *β* values of 485,577 CpGs. The performance of the models was evaluated on the test set using the ROC curve. Among the six algorithms, the logistic regression analysis showed the best classification performance: the sensitivity and specificity of one panel consisting of seven CpGs in *TFAP2A*, *TBX15*, *PRR15*, *HOXA11*, *PDGFRA*, *TOX2*, and *PHF11* genes (Fig. 1a) were 87.0 and 83.3% in the test set, respectively. The area under the receiver operating characteristic (ROC) curve was equal to 0.87 (95% confidence interval = 073–0.96, *p* < 0.001; Fig. 1b).

### Methylation profiling was different between bronchial washing and surgically resected tumor tissue

**Fig. 2:**
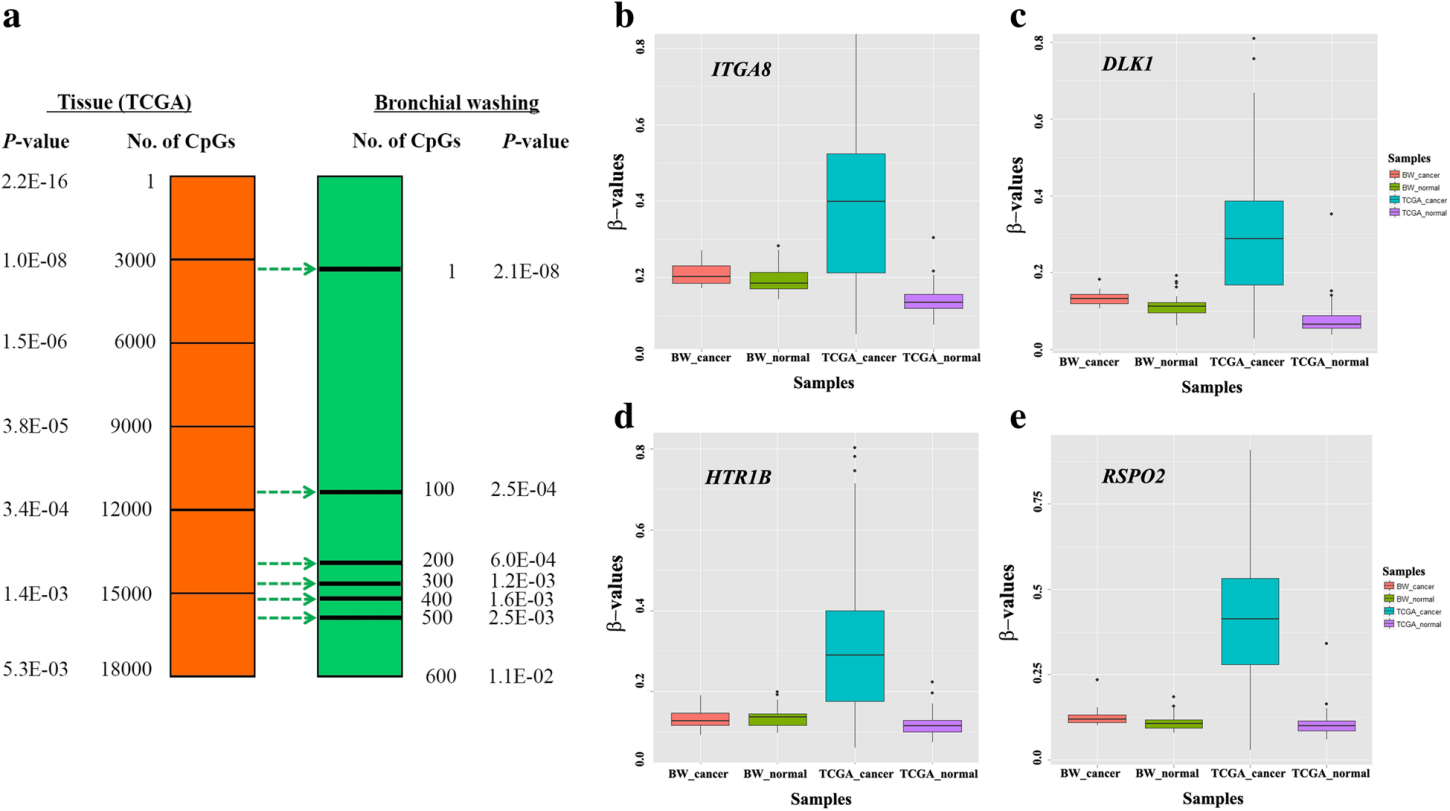
Comparison of the most highly methylated CpGs between TCGA cohort and bronchial washings. **a** Statistical significances of top 18,000 CpGs discovered from the TCGA cohort were compared to those of top 600 CpGs from bronchial washing (*N* = 123). The number of differentially methylated CpGs was significantly higher in the TCGA cohort than that in bronchial washings. **b**–**e** Hundreds of genes were found to be significantly methylated in the TCGA cohort only. Methylation levels of representative genes important in the pathogenesis of NSCLC were compared between the TCGA cohort and bronchial washings: CpGs in *ITGA8* (**b**), *DLK1* (**c**), *HTR1B* (**d**), and *RSPO2* (**e**) genes were significantly methylated (*p* < 1.0E−07) in the TCGA cohort. The *Y*-axis represents *β* values

To understand methylation profiling between bronchial washing and surgically resected tumor tissue, we compared the number and *p* values of CpGs showing statistical significance (*p* < 1.0E−07) in 123 bronchial washings and 897 TCGA primary lung tissues (821 primary tumor tissues and 76 normal tissues). All 24 CpGs showing statistical significance in bronchial washings were found to be significantly methylated in the TCGA cohort irrespective of histologic subtypes. The number of statistically significant CpGs was much higher in the TCGA cohort than that in bronchial washings (Fig. 2a). The degree of statistical significance was also lower in bronchial washings compared to that in the TCGA cohort. *p* values of a CpG showing the strongest significance were 2.2E−16 and 2.1E−08 in the TCGA cohort and bronchial washings, respectively. Over 4000 CpGs were significantly (*p* < 1.0E−07) hypermethylated in TCGA tumor tissues compared to normal tissues. Figure 2b–e shows an example of four CpGs in *ITGA8*, *DLK*1, *HTR1B*, and *RSPO2* genes significantly hypermethylated in the TCGA cohort only. A Gene Ontology (GO) analysis showed that highly methylated genes in the TCGA cohort only were largely involved in the following: (i) positive regulation of transcription from RNA polymerase II promoter, (ii) homophilic cell adhesion via plasma membrane adhesion molecules, (iii) cell-cell signaling, and (iv) G-protein coupled receptor signaling pathway, coupled to cyclic nucleotide second messenger.

### Smoking cessation is associated with reduction of methylated CpGs in nonmalignant bronchial epithelial cells

**Fig. 3:**
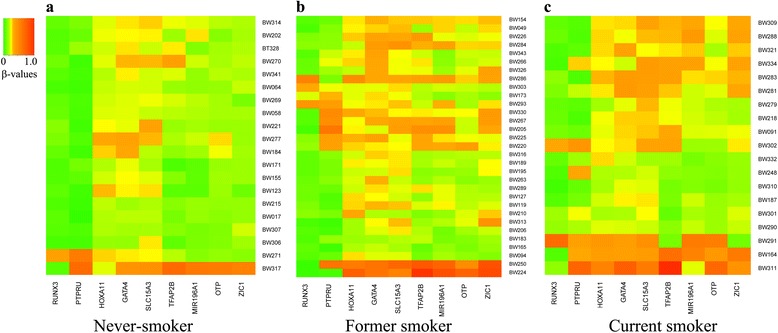
Association between methylation levels and smoking status. Methylation levels of nine CpGs were compared among never-smokers (**a**), former smokers (**b**), and current smokers (**c**). The *X*-axis represents UCSC gene names in which nine CpGs are present. The *Y*-axis represents sample identification numbers. Methylation levels are represented in a gradation of colors from green (0%) to yellow (30%) to red (100%)

**Fig. 4:**
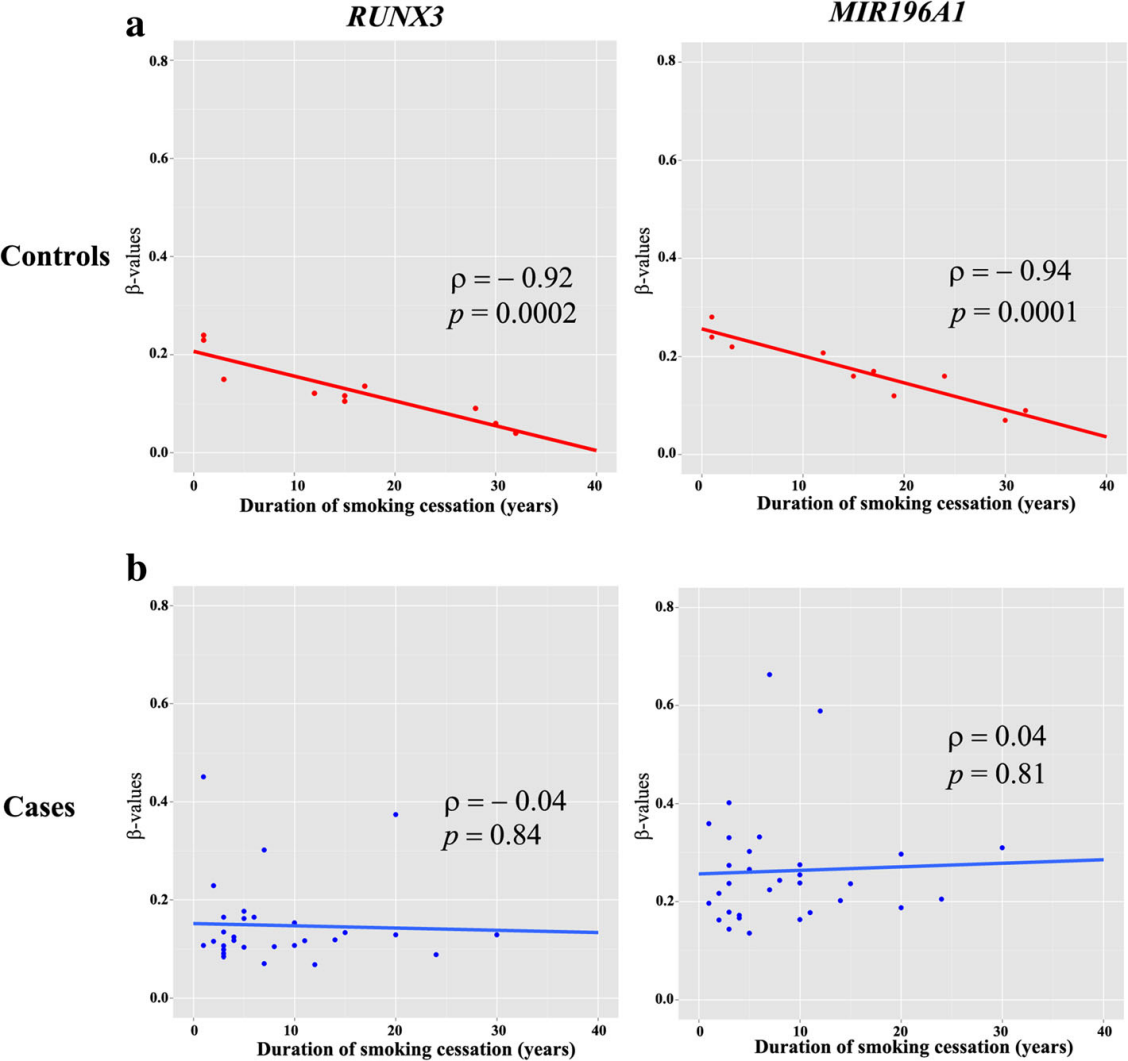
Relationship between DNA methylation and duration of smoking cessation. A correlation between methylation levels and duration of smoking cessation was analyzed in NSCLC patients (**a**) and controls (**b**). Methylation levels of CpGs on two genes (*RUNX3* and *MIR196A1*) of nine genes associated with pack-years of smoking were inversely associated with duration of smoking cessation in the control group. *p* values are based on Spearman’s correlation coefficient. The *X-* and *Y*-axes indicate duration of smoking cessation and *β* values, respectively

To understand the impact of smoking cessation on DNA methylation in bronchial washing, we first analyzed the association between levels of DNA methylation and three smoking-related variables: age at which smoking began, pack-years of cigarette smoking, and smoking status. The age at which smoking began was not associated with DNA methylation in bronchial washing (data not shown). However, aberrant methylation of nine CpGs in *RUNX3*, *MIR196A1*, *HOXA11*, *OTP*, *GATA4*, *PTPRU*, *SLC15A3*, *ZIC1*, and *TFAP2B* of 24 genes were found to be significantly associated with pack-years of smoking in controls (Additional file 5: Table S3). In addition, methylation patterns of these nine CpGs were different according to smoking status (Fig. 3). Methylation levels (*β* values) of seven of these nine CpGs were not associated with duration after smoking cessation in NSCLC or control group (data not shown). However, an inverse association was found between duration of smoking cessation and methylation levels of two CpGs in *RUNX3* and *MIR196A1* genes in the control group (Spearman’s correlation analysis; Fig. 4a), but not in NSCLC patients (Fig. 4b), suggesting that a decrease in methylation level by smoking cessation might not occur after cancer has formed. Multiple linear regression analysis in the control group showed an inverse relationship of methylation levels of CpG loci on *RUNX3* (*p* = 0.02) and *MIR196A1* genes (*p* = 0.006) with duration of smoking cessation, after adjusting for pack-years of smoking (Table 1).

**Table 1:** Multivariate regression analysis (adjusted for pack-years of smoking) for the association between DNA methylation and smoking cessation in former smokers

Parameter	Cases (*N* = 31)		Controls (*N* = 10)	
	*t* value^a^	Pr > |*t*|	*t* value	Pr > |*t*|
(1) *RUNX3*	−0.32	0.75	−3.30	0.02
(2) *MIR196A1*	−0.30	0.76	−4.15	0.006

^a^The *t* value represents *t*-statistic

## Discussion

Bronchoscopic examination for lung cancer diagnosis has been developed to overcome the limitation of sputum cytology. Several groups have reported aberrant methylation of CpG islands at the promoter region of tumor suppressor genes including *p16*, *RASSF1A*, *RARB2*, and *APC* in bronchial aspirate or sputum [3–9]. However, aberrant methylation does not indicate that methylation is tumor-specific since aberrant methylation can occur in bronchial epithelial cells due to aging. Age-related methylation has been reported for several genes, including *ER*, *N33*, *MYOD*, thrombospondin-4, and *IGF2* [18–22]. Epigenome-wide analysis has also shown changes in DNA methylation with age. Florath et al. [23] have analyzed DNA methylation using 450K array in blood DNA of 965 participants aged between 50 and 75 years and identified 65 novel CpG sites associated with aging. Bell et al. [24] have also reported age-related methylation changes in a healthy aging population at genome-wide level. Six of 31 CpGs with significant methylation in the univariate analysis of the present study showed age-related methylation in 53 controls and 70 NSCLCs (Additional file 4: Figure S2).

The number of CpGs showing statistical significance (Bonferroni-corrected *p* < 0.05) was less in bronchial washings compared to that in the TCGA cohort [25, 26] and bronchial biopsy [27]. CpGs of well-known genes such as *RARβ2*, *CDH13*, and *p16* were not included in our final CpG list since they did not meet the Bonferroni significance threshold. The small number of CpGs with statistically significant results in bronchial washings compared to the TCGA cohort might be due to contamination of normal bronchial epithelial cells during bronchial washing. Another possibility is that invasive lung cancer cells might undergo more molecular changes than bronchial epithelial cells. In addition to 24 CpGs, hundreds of CpGs were found to be significantly hypermethylated in the TCGA cohort, supporting the concept of field cancerization [28]. Widespread cellular and molecular changes during transformation of a precancerous lesion into a cancerous lesion in airway epithelia exposed to carcinogens might also occur in lung tumor tissues. Abnormal methylation found in the TCGA cohort does not mean it has diagnostic value as it can be tumor related, inflammatory cell related, or stromal response related. However, 24 CpGs that were significantly methylated in our bronchial washing samples also showed significant methylation in the TCGA cohort, suggesting that abnormal methylation found in bronchial washing might reflect some methylation changes found in tumor tissue.

A positive relationship between aberrant methylation of genes and exposure to tobacco smoke has been reported by a few groups. For example, aberrant methylation of *p16* is associated with smoking duration in primary NSCLC [29]. It was induced by tobacco-specific carcinogen, 4-methylnitrosamino-1-(3-pyridyl)-1-butanone (NNK), in lung of Fischer 344 rats [30]. Aberrant methylation of *p16*, *RARβ2*, *CDH13*, and *RASSF1A* genes has also been found in specimens of bronchial epithelial cells from cancer-free heavy smokers [31]. Buro-Auriemma et al. [32] have also reported that cigarette smoking can alter methylation patterns in small airway epithelium. In this study, aberrant methylation of nine CpGs was found to be significantly associated with pack-years of smoking in bronchial washings of the control group. Among these genes, aberrant methylation of CpGs on *GATA4*, *EMX1*, and *RUNX3* genes is known to be associated with smoke exposure [33–36]. Wood smoke exposure is associated with lower percent predicted FEV1 of COPD patients in presence of aberrantly methylated *GATA4* [33]. Methylation levels of *EMX1* are associated with pack-years of smoking in gastric mucosa of healthy individuals [34]. Positive relationship of *RUNX3* methylation to smoking has also been observed in DNA from blood leukocytes and placenta [35, 36].

Smoking cessation is a priority for preventing lung cancer. Epigenetic changes after smoking cessation have recently been identified by several groups. Several distinct CpG sites showing decreased methylation with increasing time after smoking cessation in peripheral blood lymphocytes of participants from KORA S3 survey [37] and the European Prospective Investigation into Cancer and Nutrition (EPIC) cohort [14, 15] have been reported, suggesting that a reduction of DNA methylation levels after smoking cessation may occur in a CpG site-specific manner. In this study, the impact of smoking cessation on aberrant methylation of CpG islands was analyzed in bronchial washings of former smokers. Of nine CpGs that were related to pack-years of smoking, two CpGs in *RUNX3* and *MIR196A1* genes showed an inverse relationship between the duration of smoking cessation and methylation levels of CpGs in the control group after adjusting for pack-years of smoking. Aberrant methylation of *MIR196A1* has not yet been reported in human cancer. However, *RUNX3* has been reported to be methylated in lung cancer [38, 39]. Runt-related transcription factor 3 (RUNX3) is known to function as a tumor suppressor. It also plays an important role in the regulation of cell proliferation, apoptosis, angiogenesis, cell adhesion, and invasion [40–42]. Reduction in DNA methylation levels after smoking cessation was not found in bronchial washings from lung cancer patients. Accordingly, smokers are encouraged to quit smoking prior to malignant transformation of bronchial epithelial cells.

Not many genes were found to be hypermethylated in bronchial washing in this study. This might be because only well-known genes found in lung cancer tissues were analyzed by candidate gene approach in bronchial washing fluids. Aberrant methylation of *DRD5*, *PHF11*, and *TPM1* genes discovered in this study has not been known in NSCLC up to date. CpG islands in *HOXA11* have been found to be hypermethylated in invasive NSCLC [43] and adenocarcinoma in situ (AIS) [44]. *PDGFRA* has been found to be significantly methylated in patients with NSCLC [25, 26]. *GDNF* and *TFAP2A* have been reported to be highly methylated in squamous cell carcinoma of the lung [45] and in adenocarcinoma [46], respectively. TOX high mobility group box family member 2 (*TOX2*) is not methylated in normal lung cancer cells. However, it is methylated in approximately 28% of lung cancer tissues [47].

This study was limited by several factors. First, this study was conducted in a small number of former smokers. The classification performance of a CpG panel from bronchial washing and the effect of smoking cessation on DNA methylation should be demonstrated in a large cohort in the future. Second, we did not measure the relative proportion of many other cell types (macrophages, lymphocytes, neutrophils) or total cell count in bronchial washing specimens. Therefore, it was unclear how much non-bronchial epithelial cell populations contributed to the analysis of methylation. Third, all cases were early-stage lung cancer and the size of some cancer tissues was small. Thus, we could not analyze methylation patterns of cancer tissues. Methylation patterns of tumor samples and bronchial washing samples should be compared in the same patient. Fourth, the potential impact of tumor heterogeneity and differences between methylation in invasive margin vs. tumor center need to be explored as explanations for the difference seen between bronchial washings and the TCGA cohort. Finally, the sensitivity and specificity of the CpG panel might be inflated because its prediction value was obtained from the same set of patients. Accordingly, an independent set of patients should be used in the future to determine their true values.

## Conclusions

The present study suggests that methylation analysis in bronchial washing may be helpful for detecting NSCLC. Furthermore, smoking cessation in patients with benign lung diseases may result in decreased DNA methylation in a gene-specific manner.

## Additional files

Additional file 1: **Figure S1.** Validation of 450K array. (A) A quality of 450K array was first checked by analyzing measured values for predefined subsets of methylation levels (0, 33, 66, and 100%). 2-D scatter plots were produced using plotColorBias2D in the Lumi package. The plotColorBias2D function separately plots methylated (green) and unmethylated (red) probe intensities in a 2-D scatter plot, and shows the interrogated CpG sites in red and green dots based on their color channels. (B) Methylation levels obtained by the 450K array were further validated using pyrosequencing. The sequencing output shows methylation levels at a cg27364741 locus at a promoter of *OTX1* gene in cancer (top) and control sample (bottom). (C) Methylation levels at the cg27364741 locus were compared between *β* values from 450K array (*Y*-axis) and pyrosequencing (*X*-axis). Pyrosequencing was performed in 12 bronchial washing samples. Methylation levels at the cg27364741 locus were found to be higher in 450K array than in pyrosequencing. (TIF 25467 kb)

Additional file 2: **Table S1.** Clinicopathological characteristics (*N* = 123). (DOCX 17 kb)

Additional file 3: **Table S2.** Significantly methylated CpGs in bronchial washings of lung cancer. (DOCX 23 kb)

Additional file 4: **Figure S2.** Correlation coefficients for 6 CpGs showing positive correlation between patient’s age and methylation. The relationship between patient’s age and methylation levels. A correlation between methylation levels of *EMX1* (A), *ITPKA* (B), *EVX1* (C), *HIST1H2BK* (D), *HOXA9* (F), and *PRDM14* (FG) genes and patient’s age was analyzed in 70 NSCLC patients and 53 controls separately. *p* values were based on Spearman’s rank correlation coefficient. The *X*- and *Y*-axes indicate patient’s age and *β* values, respectively. (TIF 2963 kb)

Additional file 5: **Table S3.** The correlation between pack-years of smoking and DNA methylation in 53 control groups. (DOCX 15 kb)

## Abbreviations

AUC: Area under the curve
DNMT: DNA methyltransferase
GO: Gene Ontology
NSCLC: Non-small cell lung cancer
TCGA: The Cancer Genome Atlas
TNM: Tumor-node-metastasis
