# Supplementary figures and images for: Genome-wide analysis of DNA methylation in bronchial washings
Source: Clin Epigenetics. 2018 May 18;10:65. doi: 10.1186/s13148-018-0498-8 (PMC5960087; doi:10.1186/s13148-018-0498-8)

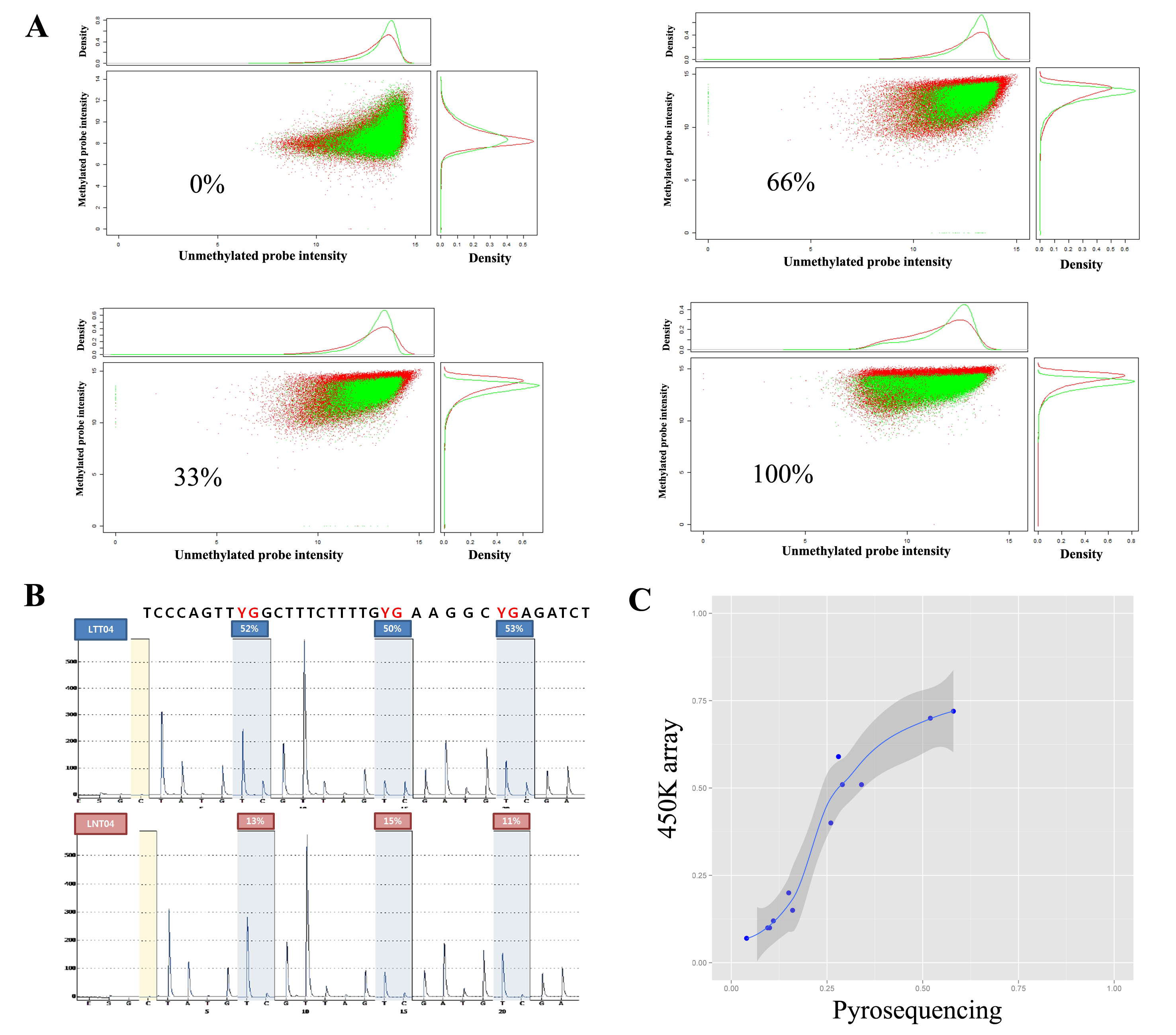

Supplement: Supplementary file 1 — Figure S1. Validation of 450K array. (A) A quality of 450K array was first checked by analyzing measured values for predefined subsets of methylation levels (0, 33, 66, and 100%). 2-D scatter plots were produced using plotColorBias2D in the Lumi package. The plotColorBias2D function separately plots methylated (green) and unmethylated (red) probe intensities in a 2-D scatter plot, and shows the interrogated CpG sites in red and green dots based on their color channels. (B) Methylation levels obtained by the 450K array were further validated using pyrosequencing. The sequencing output shows methylation levels at a cg27364741 locus at a promoter of OTX1 gene in cancer (top) and control sample (bottom). (C) Methylation levels at the cg27364741 locus were compared between β values from 450K array (Y-axis) and pyrosequencing (X-axis). Pyrosequencing was performed in 12 bronchial washing samples. Methylation levels at the cg27364741 locus were found to be higher in 450K array than in pyrosequencing. (TIF 25467 kb) [file 13148_2018_498_MOESM1_ESM.tif]

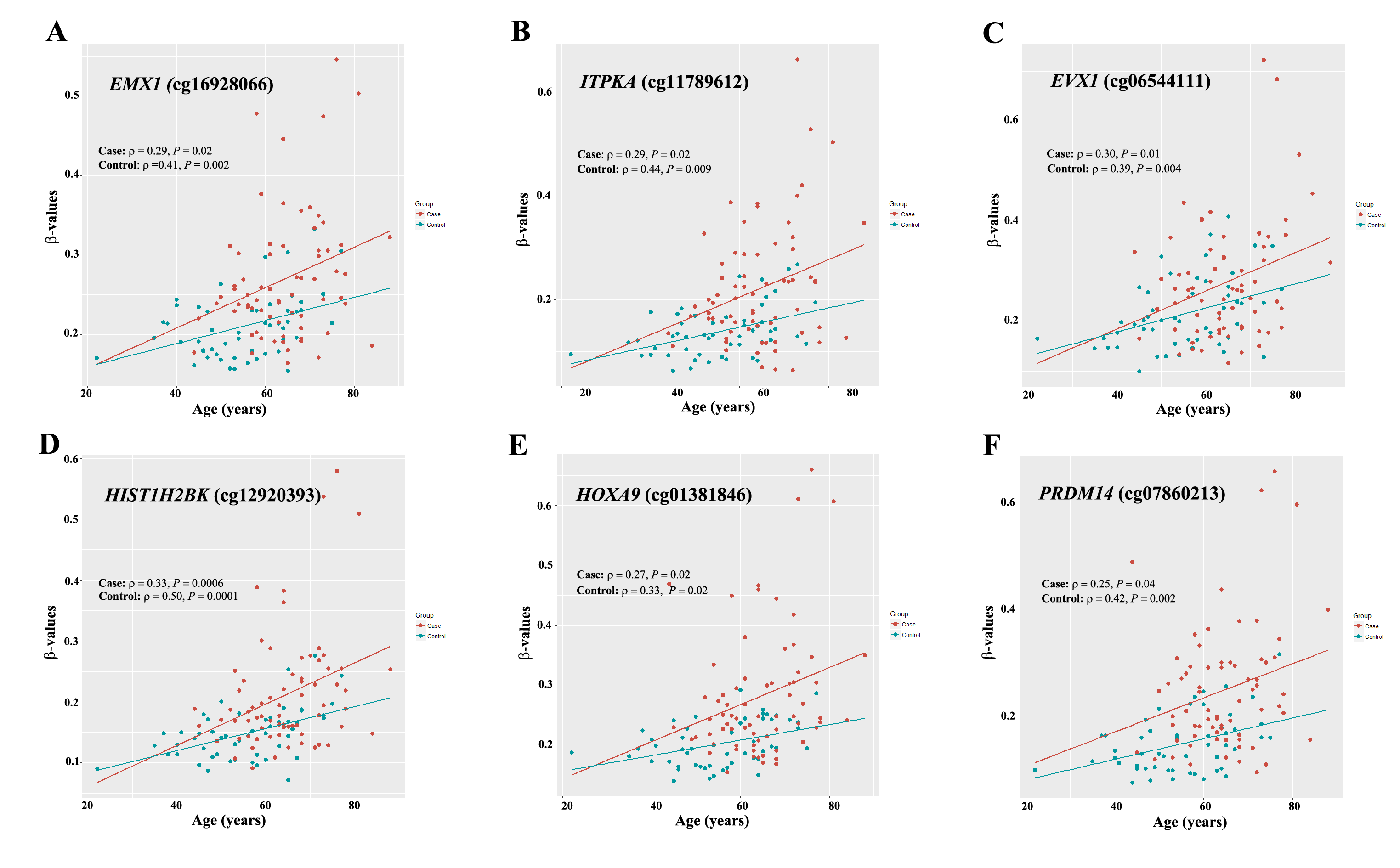

Supplement: Supplementary file 4 — Figure S2. Correlation coefficients for 6 CpGs showing positive correlation between patient’s age and methylation. The relationship between patient’s age and methylation levels. A correlation between methylation levels of EMX1 (A), ITPKA (B), EVX1 (C), HIST1H2BK (D), HOXA9 (F), and PRDM14 (FG) genes and patient’s age was analyzed in 70 NSCLC patients and 53 controls separately. p values were based on Spearman’s rank correlation coefficient. The X- and Y-axes indicate patient’s age and β values, respectively. (TIF 2963 kb) [file 13148_2018_498_MOESM4_ESM.tif]
