# Supplementary material for: Genome-wide analysis of DNA methylation in bronchial washings
Source: Clin Epigenetics. 2018 May 18;10:65. doi: 10.1186/s13148-018-0498-8 (PMC5960087; doi:10.1186/s13148-018-0498-8)
Supplement: Supplementary file 2 — Table S1. Clinicopathological characteristics (N = 123). (DOCX 17 kb) [file 13148_2018_498_MOESM2_ESM.docx]

**Additional file 2: Table S1 Clinicopathological characteristics (N=123)**

| Variables | Controls (N=53) | Cases (N=70) | p-value |
| --- | --- | --- | --- |
| Age^a^ | 55 ± 12 | 64 ± 9 | <0.0001 |
| Sex |  |  | 0.02 |
| Men | 29 | 52 |  |
| Women | 24 | 18 |  |
| Smoking status |  |  | 0.007 |
| Never | 27 | 20 |  |
| Former | 10 | 31 |  |
| Current | 12 | 19 |  |
| Pack-years of smoking^a^ | 13 ± 20 | 30 ± 32 | 0.0007 |
| Age of starting smoking^a^ | 23 ± 7 | 27 ± 8 | 0.50 |
| Duration of smoking cessation^a^ | 15 ± 12 | 8 ± 7 | 0.10 |
| Histology |  |  |  |
| Adenocarcinoma |  | 48 |  |
| Squamous cell carcinoma |  | 19 |  |
| Others |  | 3 |  |
| Pathologic stage |  |  |  |
| IA |  | 39 |  |
| IB |  | 8 |  |
| IIA |  | 4 |  |
| IIB |  | 7 |  |
| IIIA |  | 12 |  |
| Site |  |  |  |
| RUL |  | 20 |  |
| RML |  | 3 |  |
| RLL |  | 11 |  |
| LUL |  | 28 |  |
| LLL |  | 8 |  |

Abbreviations: RUL, right upper lobe; RML, right middle lobe; RLL, right lower lobe;

LUL, left upper lobe; LLL, left lower lobe

^a^Values indicate mean ± standard deviation
