# Supplementary material for: Genome-wide analysis of DNA methylation in bronchial washings
Source: Clin Epigenetics. 2018 May 18;10:65. doi: 10.1186/s13148-018-0498-8 (PMC5960087; doi:10.1186/s13148-018-0498-8)
Supplement: Supplementary file 3 — Table S2. Significantly methylated CpGs in bronchial washings of lung cancer. (DOCX 23 kb) [file 13148_2018_498_MOESM3_ESM.docx]

**Additional file 3: Table S2. Significantly methylated CpGs in bronchial washings of lung cancer**

| TargetID | UCSC_REFGENE_NAME | UCSC_CPG_ISLANDS_NAME | UCSC_REFGENE_GROUP | RELATION_TO_  UCSC_CPG_ISLAND | MAPINFO |
| --- | --- | --- | --- | --- | --- |
| cg09537620 | PAX6 | chr11:31825743-31826967 | Body | Island | 31826574 |
| cg24005685 | PITX2 | chr4:111549879-111550203 | Body | Island | 111549880 |
| cg01381846 | HOXA9 | chr7:27203915-27206462 | 1stExon | Island | 27204785 |
| cg06544111 | EVX1 | chr7:27282086-27283136 | TSS1500 | N_Shore | 27281344 |
| cg09782540 | TFAP2A | chr6:10398573-10398812 | Body | Island | 10398698 |
| cg20683765 | GDNF | chr5:37836747-37840726 | 5'UTR;TSS1500;5'UTR | N_Shore | 37835168 |
| cg01137401 | ZIC4 | chr3:147108511-147111703 | Body | N_Shore | 147107349 |
| cg19590532 | RUNX3 | chr1:25255527-25259005 | Body | Island | 25255920 |
| cg01452847 | MIR196A1 | chr17:46710812-46711419 | TSS1500 | Island | 46711341 |
| cg03045635 | DRD5 | chr4:9783035-9784960 | TSS200 | Island | 9783198 |
| cg25903072 | TPM1 | chr15:63334233-63336063 | TSS200 | Island | 63334703 |
| cg21908110 | RASSF1 | chr3:50377803-50378540 | Body | N_Shore | 50377755 |
| cg24988255 | HOXA11 | chr7:27225050-27225629 | Body;TSS1500 | Island | 27225396 |
| cg07336617 | OTP | chr5:76934581-76935296 | TSS1500 | Island | 76935080 |
| cg12920393 | HIST1H2BK | chr6:27107138-27107394 | 3'UTR;1stExon | Island | 27107145 |
| cg24646414 | GATA4 | chr8:11565217-11567212 | 5'UTR | Island | 11565725 |
| cg16928066 | EMX1 | chr2:73143055-73148260 | Body | Island | 73147814 |
| cg11789612 | ITPKA | chr15:41793562-41795305 | Body | Island | 41794412 |
| cg07860213 | PRDM14 | chr8:70981873-70984888 | Body | Island | 70982039 |
| cg26884027 | PTPRU | chr1:29585897-29586598 | Body | Island | 29586418 |
| cg21992250 | SLC15A3 | chr11:60718428-60718888 | 1stExon;Body | Island | 60718709 |
| cg14750948 | ZIC1 | chr3:147130342-147130577 | Body | Island | 147130477 |
| cg27260772 | TFAP2B | chr6:50791110-50791573 | Body | Island | 50791202 |
| cg11319389 | TOX2 | chr20:42543097-42545137 | TSS200;Body;TSS200 | Island | 42544648 |
| cg18497508 | FOXP4 | chr6:41528266-41528900 | 5'UTR | Island | 41528345 |
| cg22924269 | PHF11 | chr13:50070022-50070719 | TSS1500;TSS200 | N_Shore | 50069722 |
| cg11182225 | TAC1 | chr7:97361132-97363018 | TSS1500 | N_Shore | 97360615 |
| cg23289079 | PRDM6 | chr5:122430676-122431443 | Body | N_Shore | 122430476 |
| cg14872260 | PDGFRA | chr4:55096185-55100331 | TSS200 | N_Shore | 55095209 |
| cg05940231 | TBX15 | chr1:119531991-119532196 | TSS200 | Island | 119532189 |
| cg16021428 | ADCY2 | chr5:7394986-7397022 | TSS1500 | Island | 7395395 |

Abbreviations: UCSC_REFGENE_NAME, Gene name (UCSC);

UCSC_CPG_ISLANDS_NAME, CpG island name (UCSC);

UCSC_REFGENE_GROUP, Gene region feature category (UCSC);

Relation_to_UCSC_CpG_Island, Relationship to Canonical CpG Island, Shores - 0-2 kb from CpG island; Shelves - 2-4 kb from CpG island.

MAPINFO, Coordinates - genome build 37
