# Supplementary material for: Genome-wide analysis of DNA methylation in bronchial washings
Source: Clin Epigenetics. 2018 May 18;10:65. doi: 10.1186/s13148-018-0498-8 (PMC5960087; doi:10.1186/s13148-018-0498-8)
Supplement: Supplementary file 5 — Table S3. The correlation between pack-years of smoking and DNA methylation in 53 control groups. (DOCX 15 kb) [file 13148_2018_498_MOESM5_ESM.docx]

**Additional file 5: Table S3. The correlation between pack-years of smoking and**

**DNA methylation in 53 control groups**

| TargetID | UCSC gene names | Control |
| --- | --- | --- |
| cg24988255 | HOXA11 | ρ = 0.39, *p* = 0.006 |
| cg07336617 | OTP | ρ = 0.50, *p* = 0.0003 |
| cg19590532 | RUNX3 | ρ = 0.29, *p* = 0.04 |
| cg01452847 | MIR196A1 | ρ = 0.46, *p* = 0.001 |
| cg24646414 | GATA4 | ρ = 0.44, *p* = 0.002 |
| cg26884027 | PTPRU | ρ = 0.51, *p* = 0.0002 |
| cg21992250 | SLC15A3 | ρ = 0.47, *p* = 0.0007 |
| cg14750948 | ZIC1 | ρ = 0.33, *p* = 0.02 |
| cg27260772 | TFAP2B | ρ = 0.29, *p* = 0.04 |

* *P*-values are based on Spearman's rank correlation coefficient.
